# Kinetics of Circulating MicroRNAs in Response to Cardiac Stress in Patients With Coronary Artery Disease

**DOI:** 10.1161/JAHA.116.005270

**Published:** 2017-07-27

**Authors:** Felix Jansen, Lisa Schäfer, Han Wang, Theresa Schmitz, Anna Flender, Robert Schueler, Christoph Hammerstingl, Georg Nickenig, Jan‐Malte Sinning, Nikos Werner

**Affiliations:** ^1^ Department of Internal Medicine II Rheinische Friedrich‐Wilhelms University Bonn Germany

**Keywords:** coronary atherosclerosis, coronary heart disease risk, microRNA, Biomarkers, Basic Science Research, Translational Studies, Coronary Artery Disease

## Abstract

**Background:**

Circulating microRNAs (miRNAs/miRs) are regulated in patients with coronary artery disease. The impact of transient coronary ischemia on circulating miRNA levels is unknown. We aimed to investigate circulating miRNA kinetics in response to cardiac stress in patients with or without significant coronary stenosis.

**Methods and Results:**

Eighty of 105 screened patients with stable coronary artery disease underwent dobutamine stress echocardiography before coronary angiography. Nine circulating vascular miRNAs (miRNA‐21, miRNA‐26, miRNA‐27a, miRNA‐92a, miRNA‐126‐3p, miRNA‐133a, miRNA‐222, miRNA‐223, and miRNA‐199‐5p) were quantified in plasma by reverse transcription polymerase chain reaction before, immediately after, and 4 and 24 hours after dobutamine stress echocardiography. Quantitative polymerase chain reaction revealed increased miRNA‐21, miRNA‐126‐3p, and miRNA‐222 levels at 24 hours after dobutamine stress echocardiography in all patients. On coronary angiography, significant coronary artery stenoses (>80% diameter stenosis) were found in 41 patients. Stratifying patients according to the prevalence of significant stenoses, patients with stenosis showed an increase of circulating miRNA‐21, miRNA‐126‐3p, and miRNA‐222 in response to cardiac stress. In patients without significant stenoses (<50% diameter stenosis), miRNA‐92a levels gradually increased in response to cardiac stress.

**Conclusions:**

miRNAs are distinctly released into the circulation in response to cardiac stress depending on the prevalence of significant coronary stenoses.

**Clinical Perspective**

**What Is New?**

- Previous studies have shown the potential diagnostic value of circulating microRNAs in patients with coronary artery disease. Our study extends these findings by demonstrating that patients with coronary artery disease show specific circulating microRNA expression profiles in response to cardiac stress depending on the prevalence of significant coronary stenoses.

**What Are the Clinical Implications?**

- Based on our findings, one may speculate that circulating microRNAs might be a useful tool for discriminating patients with coronary artery disease according to the prevalence of a significant stenosis.

## Introduction

MicroRNAs (miRNAs) are small (22‐nucleotide) noncoding RNAs that regulate gene expression at the post‐transcriptional level by binding to the target mRNA, leading either to mRNA degradation or to translational repression.^1^ miRNAs have emerged as important regulators of several physiological and pathophysiological processes in cardiovascular health and disease.^2^ Besides their intracellular function, many studies have demonstrated that miRNAs can be exported or released by cells and circulate within the blood in a remarkably stable form.^3^ As circulating miRNA levels are altered depending on the prevalence or state of diseases, miRNA might be a valuable biomarker for diagnosing and monitoring cardiovascular and other diseases.^4^, ^5^

Coronary artery disease (CAD) and acute coronary syndromes lead to alterations in circulating vascular and myocardial miRNA expression profiles. In patients with stable CAD, circulating levels of vascular miRNA‐126, miRNA‐17, and miRNA‐92a are significantly reduced compared with that in healthy persons.^6^ More recently, alterations in an miRNA panel including miR‐132, miR‐150, and miR‐186 facilitated the diagnostic pathway in unstable angina pectoris.^7^ In acute myocardial infarction, predominantly miRNAs of myocardial origin are released into the circulation such as miRNA‐1, miRNA‐208a/b, and miRNA‐499.^8^, ^9^, ^10^, ^11^

Transient coronary ischemia locally and systemically impairs vascular endothelial function and myocardial perfusion. However, whether vascular or myocardial miRNAs are selectively released into the circulation in response to transient coronary ischemia is unknown.

In this study, we explored miRNA kinetics in patients with stable CAD with or without significant coronary stenosis in response to cardiac stress induced by dobutamine stress echocardiography (DSE).

We present evidence that vascular and myocardial miRNAs are distinctly released into the circulation after DSE in patients with angiographically confirmed significant coronary stenosis.

Our findings suggest that circulating miRNAs might be useful biomarkers to discriminate patients with known CAD and typical symptoms according to the prevalence of significant coronary stenosis.

## Methods

### Patient Population

**Figure 1:**
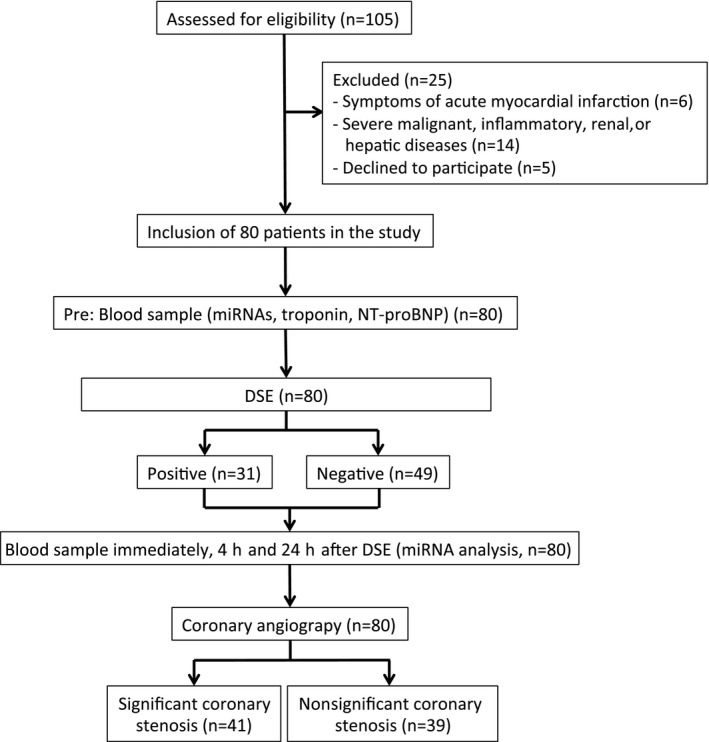
Study flow. According to the study protocol, study participants underwent dobutamine stress echocardiography (DSE) on the day before quantitative invasive coronary angiography. Blood was drawn before, immediately after, and 4 and 24 hours after DSE. miRNA indicates microRNA; NT‐proBNP, N‐terminal pro–brain natriuretic peptide.

Patient characteristics and preprocedural and postprocedural management have been recently published.^12^ Between March 2011 and March 2012, 105 patients with previously documented stable CAD and indication for coronary angiography because of typical symptoms were screened for inclusion into this study. Among those, 6 patients with clinical presentation of acute or subacute myocardial infarction were excluded from the study. Another 14 patients with malignant, inflammatory diseases or severe hepatic or renal dysfunction were also excluded from the study. Five patients declined to participate. Finally, the remaining 80 patients were included in the study after written informed consent. The day before invasive coronary angiography, all individuals underwent DSE with serial measurement of miRNA and biomarker levels (Figure 1). The study has been registered (Deutsches Register Klinischer Studien, Freiburg, DRKS‐ID: DRKS00000737) and was approved by the local ethics committee of the University of Bonn (No. 169/10).

### Dobutamine Stress Protocol

Study participants were asked to avoid agents that could antagonize the effects of dobutamine (eg, β‐blockers) before DSE. Intravenous dobutamine infusion was started at 5 μg/kg per minute and increased by 10 μg/kg per minute at 3‐minute intervals to a maximum of 40 μg/kg per minute until 85% of maximum age‐predicted heart rate was reached.^13^ If the peak maximum heart rate was not reached with dobutamine, 1.0 mg of atropine was given, if the patient had no contraindication. The test was terminated before the maximum heart rate was reached if the patient developed significant ischemia or arrhythmia, and, if necessary for clinical stability, β‐blockers were administered to reverse these conditions.

All DSE were conducted using a Phillips iE33 system with X5‐1 transducer (Philips Medical Systems). Echocardiographic imaging was performed from the parasternal long‐ and short‐axis, apical long‐axis, and apical 4‐ and 2‐chamber view. Images were recorded in resting condition, after each dobutamine infusion increase, at peak stress, and after cessation of stress. In the presence of obvious or suspected dyssynergy, a complete echocardiographic examination was performed and recorded from all employed approaches to allow optimal documentation of the presence and extent of myocardial ischemia. Analysis and scoring of the echocardiographic studies were performed using a 17‐segment model of the left ventricle and a 4‐grade scale of regional wall motion analysis (1=normal, 2=hypokinetic, 3=akinetic, and 4=dyskinetic).

### Coronary Angiography

Coronary angiography was performed in all individuals according to the guidelines of the European Society of Cardiology. A significant stenosis was defined as diameter stenosis ≥80% and no significant stenosis was defined as diameter stenosis <50% (as determined by quantitative coronary angiography) in a major native epicardial coronary artery with a diameter of ≥2.5 mm. The interventional strategy was left to the discretion of the treating operator, who was blinded to MP and biomarker results and standard practice in accordance with the European Society of Cardiology guidelines for myocardial revascularization.^14^

### miRNA Measurement in Plasma

#### Preparation of blood samples

Venous blood was drawn under sterile conditions from the cubital vein and was buffered using EDTA. Additional blood samples for routine analyses were obtained. Blood was centrifuged at 1500*g* for 15 minutes followed by centrifugation at 13 000*g* for 2 minutes to generate platelet‐deficient plasma.^15^ Platelet‐deficient plasma was stored at −80°C until miRNA levels were analyzed. After centrifugation, samples were transferred to RNase/DNase‐free tubes and stored at −80°C until miRNA levels were analyzed.

#### RNA isolation

RNA was isolated from plasma using TRIzol‐based miR isolation protocol as previously described.^6^, ^15^ Briefly, for each patient, 250 μL of plasma were diluted in 750 μL TRIzol LS (Life Technologies). *Caenorhabditis elegans* miR‐39 (cel‐miR‐39, 5 nmol/L, Qiagen) was spiked in TRIzol for normalization of miR content as described.^6^, ^15^ To increase the yield of small RNAs, the RNA was precipitated in ethanol at −20°C overnight with glycogen (Invitrogen).

#### Quantification of miRNAs by Quantitative Polymerase Chain Reaction

RNA was quantified using a Nanodrop spectrophotometer (Nanodrop Technologies Inc). Ten nanogram of the total RNA was reversely transcribed using a TaqMan microRNA reverse transcription kit (Applied Biosystems) according to the manufacturer's protocol. miRNA‐21, miRNA‐26, miRNA‐27a, miRNA‐92a, miRNA‐126‐3p, miRNA‐133a, miRNA‐222, miRNA‐223, and miRNA‐199‐5 were detected using TaqMan microRNA assays (Applied Biosystems) on a 7500 HT Real‐Time PCR machine (Applied Biosystems). Cel‐miR‐39 was used as an endogenous control. For all miRs, a Ct value >40 was defined as undetectable. Delta Ct method was used to quantify relative miRNA expression. Values were normalized to cel‐miR‐39 and are expressed as 2^−dct^ log 10. For all polymerase chain reaction experiments, samples were run in triplicates. The cel‐miR‐39 measurements across samples were stable and did not exceed 1 Ct value in >99% of all samples.

### Statistical Analysis

Data are presented as mean±SD if normally distributed or as median and interquartile range (interquartile range: quartile 1–quartile 3) if not normally distributed. Continuous variables were tested for normal distribution with the use of the Kolmogorov–Smirnov test. Categorical variables are given as frequencies and percentages. For continuous variables, Student *t* or Mann–Whitney *U* test was used for comparison between 2 groups. For the comparison of >2 groups, the 1‐way ANOVA with Bonferroni's correction for multiple comparisons was used.

All tests were 2‐sided, and a *P* value <0.05 was considered statistically significant. Statistical analyses were conducted with PASW Statistics version 22.0.0 64‐bit (IBM Corporation) and MedCalc version 11.1.1.0 (MedCalc Software) and GraphPad Prism 5.

All authors attest to the accuracy and completeness of the data and all analyses and confirm that the study was conducted according to the protocol.

## Results

### Baseline Characteristics

From the initially screened 105 patients with stable CAD, 80 patients (mean age, 66.9±10.9 years; 71% men) were enrolled from March 2011 to March 2012 in this study (Figure 1). Preprocedural and postprocedural management of the patients has been recently published.^12^ All patients underwent DSE on the day before coronary angiography. Quantitative invasive coronary angiography revealed a significant stenosis (as determined by quantitative coronary angiography as ≥80% diameter stenosis of ≥1 major epicardial coronary artery with a diameter ≥2.5 mm) in 41 patients (89.5±4.3% diameter stenosis). Baseline characteristics of the study participants are shown according to the prevalence of a significant stenosis in Table 1. Patient groups did not differ regarding cardiovascular risk factors and baseline levels of miRNA‐21, miRNA‐26, miRNA‐27a, miRNA‐126‐3p, miRNA‐133, miRNA‐199a, miRNA‐222, and miRNA‐223. Expression of baseline circulating miRNA‐92a was significantly higher in patients with significant coronary stenosis.

**Table 1:** Baseline Characteristics According to Prevalence of Significant Coronary Artery Stenosis

Characteristics	Total (N=80)	Nonsignificant Stenosis (n=39)	Significant Stenosis (n=41)	*P* Value
Age, y	66.9±10.9	67.4±10.5	66.4±11.4	0.67
Sex				0.70
Female	23 (28.8)	12 (30.8)	11 (26.8)	
Male	57 (71.3)	27 (69.2)	30 (73.2)	
Cardiovascular risk factors				
Arterial hypertension	80 (100)	39 (100)	41 (100)	NA
Hyperlipoproteinemia	66 (82.5)	32 (82.1)	34 (82.9)	0.92
Diabetes mellitus	19 (23.8)	6 (15.4)	13 (31.7)	0.09
Family history of CAD	25 (31.3)	14 (35.9)	11 (26.8)	0.38
Smoking	29 (36.3)	11 (28.2)	18 (43.9)	0.14
Body mass index, kg/m^2^	27.6±4.0	27.0±4.2	28.0±3.9	0.41
Laboratory parameters				
Cholesterol, mg/dL	180.1±38.9	178.8±42.4	181.2±36.0	0.79
LDL cholesterol, mg/dL	101.7±28.1	103.2±30.3	100.2±25.8	0.65
HDL cholesterol, mg/dL	49.9±13.3	50.2±15.1	49.6±11.6	0.84
Hemoglobin, g/dL	14.6±1.5	14.5±1.6	14.7±1.3	0.54
Leucocytes, 10^9^/L	7.2±1.9	6.9±1.9	7.4±1.9	0.31
Serum creatinine, mg/dL	1.02±0.31	1.02±0.28	1.04±0.34	0.77
C‐reactive protein, mg/L	1.50 (0.78/3.93)	1.55 (0.93/4.48)	1.50 (0.68/3.18)	0.23
Medical history				
Previous myocardial infarction	28 (35.0)	10 (25.6)	18 (43.9)	0.09
Previous PCI	39 (48.8)	16 (41.0)	23 (56.1)	0.18
Previous stroke	2 (2.5)	0 (0)	2 (4.9)	0.16
COPD	7 (8.8)	6 (15.4)	1 (2.4)	0.04^a^
Peripheral arterial disease	10 (12.5)	3 (7.7)	7 (17.1)	0.21
Coronary artery disease				0.04^a^
1 Vessel	19 (23.8)	14 (35.9)	5 (12.2)	
2 Vessels	21 (26.3)	9 (23.1)	12 (29.3)	
3 Vessels	40 (50.0)	16 (41.0)	24 (58.5)	
DSE result				0.001^a^
Positive	31 (38.8)	8 (20.5)	23 (56.1)	
Negative	49 (61.2)	31 (79.5)	18 (43.9)	
Left ventricular ejection fraction, %	57.0±11.8	58.2±10.8	55.9±12.7	0.40
Medication on admission				
ACEI or ARB	66 (82.5)	32 (82.1)	34 (82.9)	0.69
β‐Blockers	43 (53.8)	18 (46.2)	25 (61.0)	0.18
Statins	45 (56.3)	18 (46.2)	27 (65.9)	0.08
Aspirin	75 (93.8)	36 (92.3)	39 (95.1)	0.60
Circulating miRNAs (log‐2dct)				
miRNA‐21	4.27±0.33	4.21±0.18	4.35±0.845	0.10
miRNA‐26a	4.35±0.22	4.36±0.18	4.34±0.26	0.80
miRNA‐27a	4.25±0.24	4.28±0.18	4.23±0.26	0.50
miRNA‐92a	3.87±0.03	3.81±0.04	3.95±0.04	0.03^a^
miRNA‐126‐3p	4.16±0.29	4.21±0.15	4.11±0.37	0.12
miRNA‐133a	4.54±0.83	4.54±1.08	4.54±0.31	0.99
miRNA‐199a‐5p	4.54±0.29	4.57±0.20	4.48±0.39	0.26
miRNA‐222	4.14±0.34	4.19±0.16	4.11±0.44	0.31
miRNA‐223	3.93±0.28	3.95±0.04	3.86±0.05	0.18

Values are number (percentage) or mean±SD. ACEI indicates angiotensin‐converting enzyme inhibitor; ARB, angiotensin receptor blocker; CAD, coronary artery disease; COPD, chronic obstructive pulmonary disease; DSE, dobutamine stress echocardiography; HDL, high‐density lipoprotein; LDL, low‐density lipoprotein; miRNA, microRNA; NA, not applicable; PCI, percutaneous coronary intervention.

a *P* ≤ 0.05.

### Circulating miRNAs in Response to Cardiac Stress

Transient coronary ischemia locally and systemically affects vascular endothelial function and myocardial oxygen supply. Circulating miRNAs have been shown to be regulated in patients with CAD and hold great potential for the use as a diagnostic and prognostic biomarker.^2^, ^6^, ^15^, ^16^ In order to explore whether circulating miRNAs are selectively released into the circulation in response to cardiac stress, 9 selected miRNAs that have been shown to be involved in several cardiovascular pathologies (miRNA‐21, miRNA‐26, miRNA‐27a, miRNA‐92a, miRNA‐126‐3p, miRNA‐133a, miRNA 199‐5p, miRNA‐222, and miRNA‐223) were quantified immediately and 4 and 24 hours after DSE in all patients.

**Figure 2:**
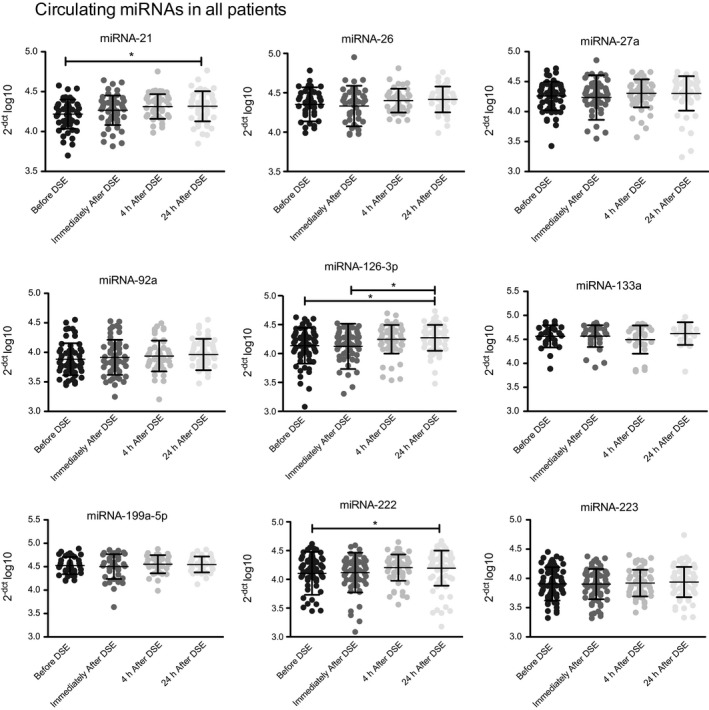
Release kinetics of circulating microRNAs (miRNAs) in patients with stable coronary artery disease in response to cardiac stress. Circulating miRNAs were analyzed before, immediately after, and 4 and 24 hours after dobutamine stress echocardiography (DSE). Delta Ct method was used to quantify relative miRNA expression. Values were normalized to cel‐miR‐39 and are expressed as 2^−[^ ^CT^ ^(micro^ ^RNA^ ^)−^ ^CT^ ^(cel‐miR‐39)]^ log10. Data are presented as mean±SEM. *P<0.05, by 1‐way ANOVA with Bonferroni's correction for multiple comparisons.

Cardiac stress induced by DSE resulted in significantly elevated levels of circulating miRNA‐21, miRNA‐126‐3p, and miRNA‐222 24 hours after DSE (Figure 2). The remaining analyzed miRNAs did not show any significant regulation after cardiac stress.

### Circulating miRNAs Stratified According to the Prevalence of Significant Coronary Stenoses

To assess whether cardiac ischemia as a result of significant coronary artery stenoses during cardiac stress is associated with selective miRNA release, we stratified the patients according to the prevalence of significant coronary stenoses (significant coronary stenosis: n=41, nonsignificant coronary stenosis: n=39). A significant stenosis was defined as diameter stenosis ≥80% determined by quantitative coronary angiography in a major native epicardial coronary artery with a diameter of ≥2.5 mm.

**Figure 3:**
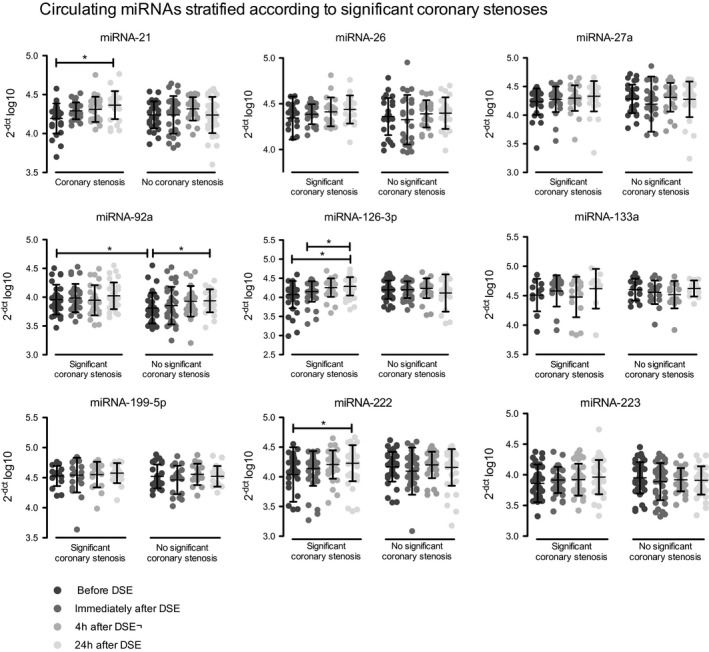
Release kinetics of circulating microRNAs (miRNAs) in patients with stable coronary artery disease according to the prevalence of significant coronary stenosis. Circulating miRNAs were analyzed before, immediately after, and 4 and 24 hours after dobutamine stress echocardiography (DSE) in patients with or without significant coronary stenosis. Delta Ct method was used to quantify relative microRNA expression. Values were normalized to cel‐miR‐39 and are expressed as 2^−[^ ^CT^ ^(micro^ ^RNA^ ^)−^ ^CT^ ^(cel‐miR‐39)]^ log10. Data are presented as mean±SEM. *P<0.05, by 1‐way ANOVA with Bonferroni's correction for multiple comparisons.

Patients with a significant coronary stenosis showed significantly increased levels of circulating miRNA‐21, miRNA‐126‐3p, and miRNA‐222 in response to cardiac stress 24 hours after DSE compared with baseline (Figure 3). The remaining analyzed miRNAs were not regulated in response to cardiac stress in patients with significant coronary stenoses (Figure 3). In patients without significant coronary stenosis, miRNA‐92a showed a significant increase 24 hours after DSE‐induced cardiac stress. The remaining analyzed miRNAs were not regulated in response to cardiac stress in patients without significant coronary stenoses (Figure 3). Furthermore, there was no significant correlation between miRNA and troponin T levels (Table 2).

**Table 2:** Correlation Analysis of miRNA and Troponin Levels

	Troponin 0 h	Troponin 1 h	Troponin 4 h	Troponin 24 h
miRNA‐21	*r*=0.27	*r*=0.189	*r*=0.264	*r*=−0.028
	*r* ^2^=0.073	*r* ^2^=0.036	*r* ^2^=0.697	*r* ^2^=0.0008
	*P*=0.849	*P*=0.484	*P*=0.073	*P*=0.839
miRNA‐26	*r*=−0.036	*r*=0.170	*r*=0.164	*r*=0.071
	*r* ^2^=0.0013	*r* ^2^=0.029	*r* ^2^=0.027	*r* ^2^=0.005
	*P*=0.813	*P*=0.529	*P*=0.306	*P*=0.653
miRNA‐27a	*r*=0.023	*r*=−0.105	*r*=0.095	*r*=−0.191
	*r* ^2^=0.0005	*r* ^2^=0.011	*r* ^2^=0.009	*r* ^2^=0.036
	*P*=0.432	*P*=0.349	*P*=0.256	*P*=0.082
miRNA‐92a	*r*=0.028	*r*=0.159	*r*=−0.042	*r*=0.000
	*r* ^2^=0.0008	*r* ^2^=0.025	*r* ^2^=0.0018	*r* ^2^=0.000
	*P*=0.417	*P*=2.265	*P*=0.386	*P*=0.498
miRNA‐126‐3p	*r*=0.017	*r*=0.278	*r*=0.206	*r*=−0.053
	*r* ^2^=0.0003	*r* ^2^=0.077	*r* ^2^=0.042	*r* ^2^=0.003
	*P*=0.443	*P*=0.132	*P*=0.058	*P*=0.327
miRNA‐133a	*r*=−0.140	*r*=0.344	*r*=0.230	*r*=−0.128
	*r* ^2^=0.020	*r* ^2^=0.118	*r* ^2^=0.053	*r* ^2^=0.016
	*P*=0.257	*P*=0.137	*P*=0.115	*P*=0.291
miRNA‐199a‐5p	*r*=0.047	*r*=0.318	*r*=0.134	*r*=−0.020
	*r* ^2^=0.002	*r* ^2^=0.101	*r* ^2^=0.018	*r* ^2^=0.0004
	*P*=0.392	*P*=0.107	*P*=0.224	*P*=0.455
miRNA‐222	*r*=0.050	*r*=0.184	*r*=0.153	*r*=−0.040
	*r* ^2^=0.0025	*r* ^2^=0.034	*r* ^2^=0.023	*r* ^2^=0.016
	*P*=0.336	*P*=0.232	*P*=0.117	*P*=0.366
miRNA‐223	*r*=0.128	*r*=0.162	*r*=0.040	*r*=−0.141
	*r* ^2^=0.016	*r* ^2^=0.026	*r* ^2^=0.0016	*r* ^2^=0.020
	*P*=0.148	*P*=0.261	*P*=0.379	*P*=0.119

Data were analyzed using Pearson correlation coefficient. miRNA indicates microRNA.

miRNAs are powerful regulators of diverse (patho)physiological processes in cardiovascular diseases.^17^ Moreover, an increasing number of studies are demonstrating that miRNAs can be detected in circulating blood, representing useful biomarkers in patients with cardiovascular disease such as coronary artery disease. However, whether circulating miRNA expression pattern might be useful to discriminate patients with known CAD and typical symptoms according to the prevalence of significant coronary stenosis is unknown.

Here, we present evidence that cardiac stress induced by DSE facilitates the release of vascular and myocardial miRNAs into the circulation in patients with stabile CAD. In addition, release kinetics of miRNAs in response to cardiac stress depend on the prevalence of significant coronary stenoses with subsequent coronary ischemia. In our study, patients with significant stenosis showed an increase of circulating levels of miRNA‐21, miRNA‐126‐3p, and miRNA‐222 after cardiac stress.

In recent years, a remarkable number of studies explored the diagnostic and prognostic potential or circulating miRNAs in patients with CAD. In one of the first studies exploring miRNAs in patients with CAD, Fichtlscherer et al^6^ showed that miRNA‐126, miRNA‐17, miRNA‐92a, and the inflammation‐associated miR‐155 were significantly reduced in patients with CAD compared with healthy controls. Furthermore, miRNA‐133a, miRNA‐134, miR‐145, miRNA‐122, and miRNA‐370 were altered in patients with CAD, even after adjustment for other cardiovascular risk factors.^18^ Exploring the prognostic potential of circulating miRNAs, 3 studies demonstrated that single miRNA levels or panel analysis were associated with cardiovascular outcome in patients after myocardial infarction.^19^, ^20^, ^21^ Of note, the expression of miRNA‐126‐3p and miRNA‐223 predicted mortality in a cohort of patients with symptomatic coronary artery disease.^19^

Several preclinical studies revealed that miRNAs can be released by various cells into the extracellular space. Predominantly miRNA‐126‐3p has been shown to be packed and liberated via microparticles or apoptotic bodies from activated or apoptotic endothelial cells.^22^, ^23^, ^24^ In contrast, endothelial release of miRNA‐126 is reduced in conditions of atheroprotective laminar shear stress.^25^ In line with our data, a recent clinical study demonstrated that miR‐126‐3p tended to be released specifically from the coronary vasculature in patients with high plaque load or vulnerable plaques.^26^

miRNA‐222 is expressed predominantly in endothelial and vascular smooth muscle cells.^27^, ^28^ Atherosclerotic vascular diseases such as peripheral artery disease or CAD are associated with reduced levels of circulating miRNA‐222.^28^, ^29^ In previous experimental studies, it was shown that miRNA‐222 was the strongest regulated miRNA between endothelial parent cells and microparticles^28^ and the second highest expressed miRNA within endothelial microparticles after miRNA‐126‐3p.^22^ Functionally, the transfer of miRNA‐222 into endothelial target cells promoted anti‐inflammatory effects by inhibiting intercellular adhesion molecule–1 expression.^28^

Whether the increase of circulating miRNA‐126‐3p and miRNA‐222 in patients with significant coronary stenoses after cardiac stress is a result of an increased local coronary miRNA release or systemic endothelial activation is unclear. Endothelial cells covering an atherosclerotic plaque significantly narrowing a coronary artery are activated and exposed to turbulent flow, leading to the release of vesicle‐ or nonvesicle‐bound vascular miRNAs such as miRNA‐126‐3p and miRNA‐222 or others into the circulation. However, as transient coronary ischemia systemically affects vascular endothelial function, increased miRNA‐126‐3p and miRNA‐222 levels could be a result of systemic endothelial activation with subsequent vascular miRNA release after cardiac stress.

miRNA‐21 has been shown to play a key role in heart failure development by regulating the extracellular signal–regulated kinase–mitogen‐activated protein kinase signaling pathway in cardiac fibroblasts, which impacts global cardiac structure and function.^30^ In a murine ischemia‐reperfusion model, myocardial miRNA‐21 was upregulated in the infarct zone and triggered fibrotic infarct remodeling by repression of phosphatase and tensin homolog.^31^ Ischemia‐induced upregulation of miRNA‐21 could represent a possible underlying mechanism of elevated circulating miRNA‐21 levels in patients with significant coronary stenosis and cardiac ischemia. In line with our findings, oscillatory stress occurring in atherosusceptible areas promotes activation and transcription of miR‐21.^32^

Whereas miRNA‐222 is mainly expressed in the vessel wall (endothelial cells and vascular smooth muscle cells), miRNA‐126‐3p has been detectable in a wide range of cells including endothelial cells,^15^, ^23^, ^33^ platelets,^34^, ^35^, ^36^ and bone marrow–derived cells.^37^, ^38^ In contrast, miRNA‐21 is ubiquitously expressed in adult human tissue.^39^

Although patients with significant coronary stenosis showed an increase of circulating miRNA‐21, miRNA‐126‐3p, and miRNA‐222, patients without stenosis revealed an increase of circulating miRNA‐92a in response to cardiac stress. As low shear stress occurs in areas of narrowing atherosclerotic plaques and turbulent flow, our findings are concordant with experimental studies demonstrating that expression of proatherogenic endothelial miRNA‐92a is induced by low shear stress conditions and exposure to oxidized low‐density lipoprotein.^40^ Why miRNA‐92a increased in response to cardiac stress only in patients without significant coronary stenosis is unclear. Given that miRNA‐92a promotes atherogenesis and vascular inflammation,^41^ one could speculate that in patients without significant coronary stenosis the endothelium releases rather damaging miRNA‐92a for self‐protection that is impaired in patients with advanced coronary artery disease.

**Figure 4:**
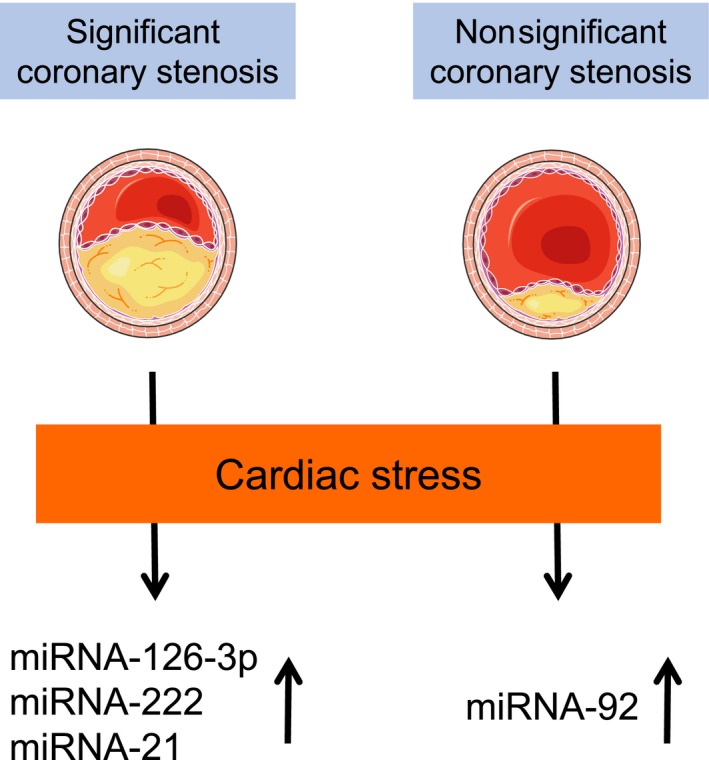
Schematic illustration: the prevalence of significant coronary stenosis modulates circulating microRNA (miRNA) expression patterns in response to cardiac stress.

Our study reveals that vasculoprotective miRNAs such as miRNA‐126 and miRNA‐222 are released in patients with cardiac ischemia, whereas atheroprone miRNA‐92a is regulated in patients without significant coronary stenosis in response to cardiac stress (Figure 4). As well as others, miRNA‐126 and miRNA‐92a are directly regulated by hemodynamic forces.^42^, ^43^ Krüppel‐like factor 2, a key player in the regulation of endothelial integrity,^44^ induces miRNA‐126 but inhibits miR‐92a expression. Alterations of Krüppel‐like factor 2 expression or other regulators of miRNA biosynthesis might be one possible underlying mechanism mediating different release kinetics of circulating miRNAs in response to cardiac stress in dependence of the coronary status.

### Study Strengths and Limitations

Previous studies have shown the potential diagnostic value of circulating miRNAs in patients with CAD.^2^ Our study extends these findings by demonstrating that patients with CAD show specific circulating miRNA expression profiles in response to cardiac stress depending on the prevalence of significant coronary stenoses. Based on our findings, one may speculate that circulating miRNAs might be a useful tool for discriminating patients with CAD according to the prevalence of a significant stenosis. Nevertheless, the study has limitations. First, only a selected number of miRNAs based on preliminary studies was analyzed and might only represent a small number of all regulated miRNAs between the patient groups. Second, we assessed circulating miRNA levels until 24 hours after DSE. miRNA expression patterns >24 hours after cardiac stress would give additional knowledge regarding release and clearance kinetics of circulating miRNA in patients with CAD. Third, we cannot exclude potential influences of dietary habits and diurnal variations on the analyzed miRNA levels. Fourth, fractional flow reserve measurements were not been performed to assess the hemodynamic relevance of coronary stenosis.

## Conclusions

The prevalence of significant coronary stenosis modulates circulating miRNA expression patterns in response to cardiac stress in patients with stable CAD.

## Sources of Funding

Werner and Jansen were supported by Deutsche Forschungsgemeinschaft (WE 4139/8‐1). Jansen was supported by the medical faculty of the Rheinische Friedrich‐Wilhelms‐University Bonn (BONFOR), the “Familie Schambach” Foundation, the German Society of Cardiology, and Deutsche Forschungsgemeinschaft (JA 2351/2‐1). Sinning was supported by research grants from the Deutsche Forschungsgemeinschaft Nachwuchsakademie (SI 1728/1‐1) and BONFOR.

## Disclosures

None.
